# Unilateral Posterior Uveitis in a Patient Receiving Nivolumab for Malignant Melanoma

**DOI:** 10.7759/cureus.43311

**Published:** 2023-08-10

**Authors:** Periklis Giannakis, Mohsan Malik, Sukaina Rashid, Eleni Vrizidou

**Affiliations:** 1 Barts and the London School of Medicine and Dentistry, Queen Mary University of London, London, GBR; 2 Adnexal Service, Moorfields Eye Hospital NHS Foundation Trust, London, GBR; 3 Medical Oncology, St. Bartholomew’s Hospital, London, GBR; 4 Ophthalmology, Queen’s Hospital, London, GBR

**Keywords:** immune-checkpoint inhibitors, immunotherapy-related adverse events, malignant melanoma metastasis, opdivo nivolumab, uveitis

## Abstract

Patients using immunotherapies like immune checkpoint inhibitors (ICIs) can develop ocular immune-related adverse effects (irAEs). Nivolumab (Opdivo^®^;Bristol-Myers Squibb, New York, NY, USA) is a commonly used ICI used to treat malignancies. A 75-year-old woman presented to our eye clinic with sudden loss in vision in the right eye. She had started nivolumab monotherapy 10 days before the onset of symptoms for the treatment of melanoma. Examination showed low visual acuity (20/170) in the right eye with few reactive cells and macular oedema and swelling in the anterior and posterior segments, respectively. Optical coherence tomography (OCT) of the right eye showed intra-retinal and sub-retinal fluid and multiple hyperreflective inner retinal round foci in the areas of inflammation. The differential diagnoses were infectious uveitis, Vogt-Koyanagi-Harada-like syndrome or masquerade retinopathy. After a full work-up, the patient was diagnosed with unilateral posterior uveitis. The patient responded to topical steroid therapy with improved vision (20/30). Uveitis is listed as an adverse effect on the prescribing list of the drug Opdivo^®^. Although not reported before, our case demonstrated unilateral involvement. We thus recommend clinicians to be wary after complaints of side effects from their patients; ocular toxicities should be considered.

## Introduction

Immunotherapies in the form of immune checkpoint inhibitors (ICIs) have been developed to treat many malignancies, including melanoma, and squamous cell carcinoma, amongst others [1]. Clinical trials have shown ICIs are efficacious, but patients report immune-related adverse effects (irAEs); at around 10-15% higher rate than patients receiving classic chemotherapy drugs. irAEs are mainly in the dermatological, gastrointestinal, hepatic, and endocrine systems [1]. However, 1% of patients may also manifest in the visual system. Unfortunately, patients with ocular irAEs are severely affected, presenting an outcome warranting further investigation [1]. We present a case of a 75-year-old woman with metastatic melanoma who suffered from unilateral uveitis due to nivolumab administration.

This article was previously presented as a poster at the Royal Society of Medicine Ophthalmology trainee and student prize meeting 2023 on June 15, 2023.

## Case presentation

A 75-year-old woman came to our eye clinic due to rapid deterioration in vision in the right eye; she denies any discomfort, trauma, or other symptoms. In her medical history, she reports having started nivolumab monotherapy to treat metastatic cutaneous melanoma (stage III) without cerebral or ocular metastatic involvement 10 days before ocular symptoms emerged.

**Figure 1:**
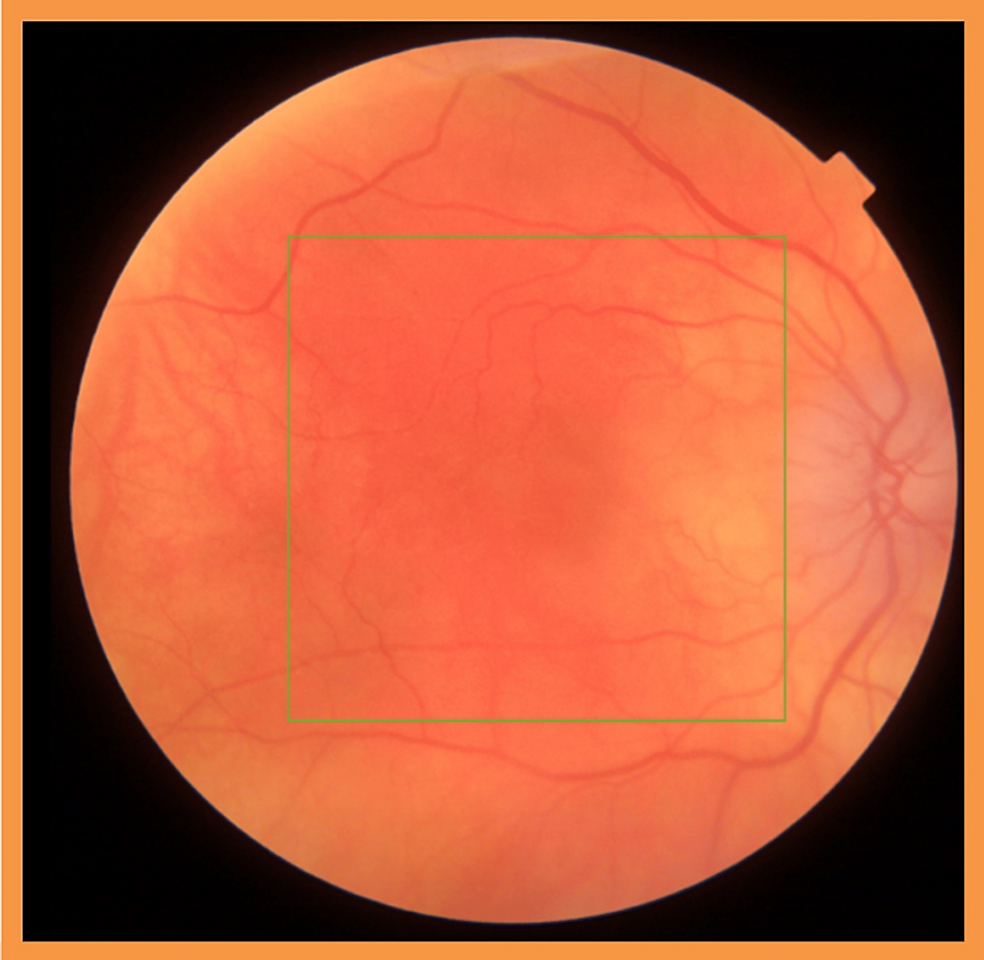
Fundus photography at the onset of ocular symptoms

On examination, we found the patient’s visual acuity was 20/170 on the right and 20/30 on the left. Anterior segment examination of the right eye revealed few reactive cells (Grade +0.5). The cornea and conjunctiva were unremarkable. The patient had bilateral reduced retinal pigmentation, in keeping with physiological variation. The right eye revealed macular oedema and optic disc swelling, with associated inflammatory cells infiltrating the vitreous (Grade +1) (Figure 1).

**Figure 2:**
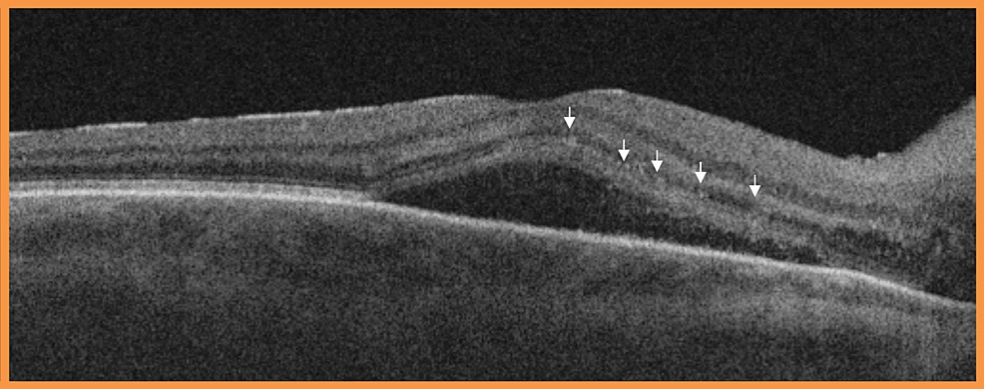
Optical coherence tomography (OCT) showed macular oedema at the onset of ocular symptoms OCT examination revealed right eye intra-retinal and sub-retinal fluid and multiple hyperreflective inner retinal round foci in inflammation (arrows)

No choroidal infiltrates were noted in either eye on optical coherence tomography (OCT)-Angiography imaging. OCT examination can be seen in Figure 2.

Given the history of immunotherapy and the unilateral nature of the presentation, we were most concerned with the possibility of infectious uveitis, Vogt-Koyanagi-Harada-like syndrome or masquerade retinopathy.

The normal retinal pigment epithelium contour and negative findings on serological and radiological testing suggested sterile inflammation due to immunotherapy prompting the accurate diagnosis of unilateral posterior uveitis.

**Figure 3:**
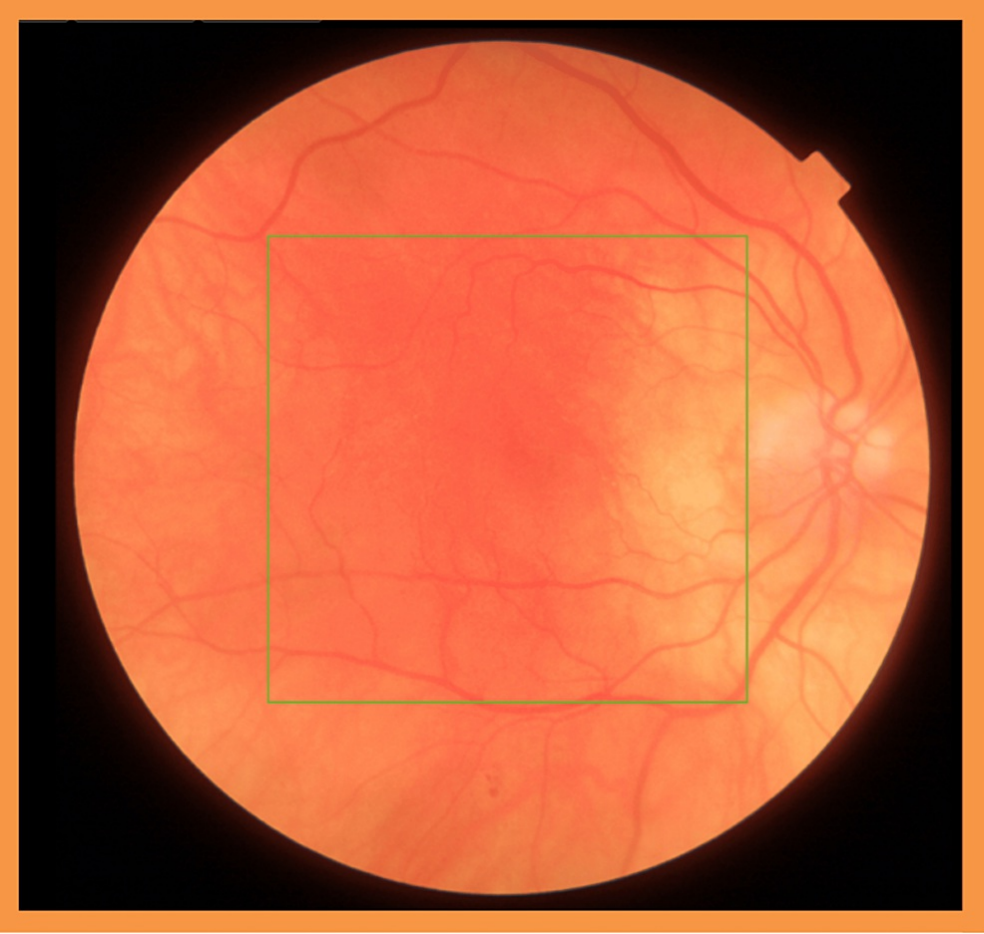
Fundus photography six months after the onset of ocular symptoms

**Figure 4:**
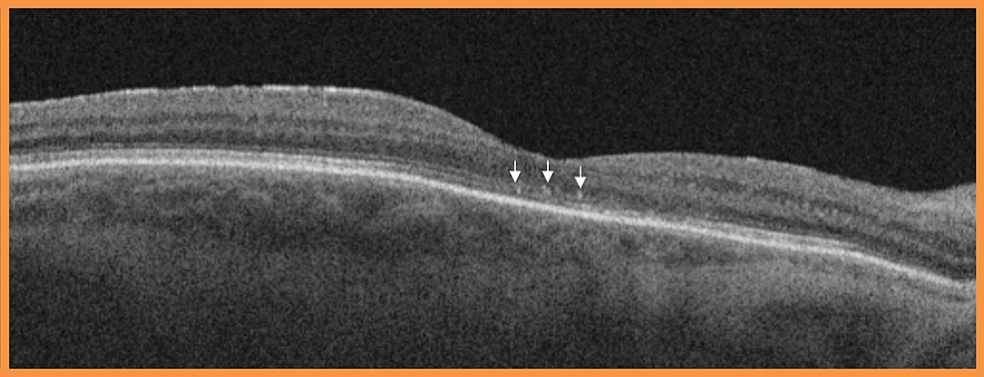
Optical coherence tomography (OCT) six months after the onset of ocular symptoms showing resolving macular oedema OCT examination revealed decrease in the previously seen intra-retinal and sub-retinal fluid and fewer hyperreflective inner retinal round foci (arrows)

The patient was prescribed g. dexamethasone 0.1% preservative-free hourly for two days, then every two hours for two days, then six times a day for seven days, then to reduce one drop per week until cessation. Following the intensive therapy with slow tapering, the vision improved to 20/30 (Figures 3, 4).

## Discussion

Nivolumab (Opdivo®; Bristol-Myers Squibb, New York, NY, USA), is a human programmed cell death-1 (PD-1) IgG4 monoclonal antibody inhibitor [2]. In cancers, activated suppressor T cells become exhausted, and instead of preventing tumour growth, they enhance it [3]. Nivolumab intercepts the interaction between PD-1 and programmed death-ligand 1 (PD-L1) and programmed death-ligand 2 (PD-L2), both ligands being upregulated in cancer conferring; as a result, increased anti-tumour immunity [3]. In the case reported here, the inhibition of the PD-1 pathway involving PD-1, PD-L1 and PD-L2 may have prompted the activated T cells to direct their function towards melanocytes, including normal ones found on the choroid [3,4].

Uveitis is listed as an adverse effect on the prescribing list of the drug Opdivo® [3]. Cases of Vogt-Koyanagi-Harada disease-like uveitis, panuveitis, and serous retinal detachment, have been reported, resulting in irreversible sight loss if left untreated [2,4,5]. Adverse events with immunotherapy are beginning to emerge, with demonstratable case reports of bilateral anterior and posterior uveitis, as well as panuveitis [5-10]. To our knowledge, that unilateral involvement event has never been reported before. We thus recommend routine screening of possible ocular symptoms in such patients to prevent blindness.

The use of topical steroids for the posterior uveitis of our patient was appropriate and followed the efficacy seen in bilateral uveitis cases secondary to nivolumab administration [5,6]. It is unclear on the mechanism by which our patient responded to topical therapy alone. Our patient was unusual to have presented with early unilateral disease, which was detected and treated. This proposes a few potential hypotheses on the mechanism of success. Firstly, posterior segment findings were possibly reactive to the anterior segment uveitis. Thereby, symptoms resolved with treatment of the anterior uveitis. Secondarily, the patient’s oncologist was informed of the potential adverse event, and the nivolumab therapy was halted, which reduced the inflammatory trigger. Finally, previous studies have found that sufficient vitreous concentration of topically administrated drugs [11], possible through the diffusion of either transvitreal or trans-uveal scleral route [12]. Although other studies have found a negligible concentration of steroids in vitreous sampling by initially giving the patient intensive treatment, this may have been sufficient in our patient's case [13].

Reports of adverse effects from nivolumab use are emerging, including bilateral uveitis and panuveitis, with unilateral posterior uveitis being a rare irAE seen in patients with aggressive melanomas treated with nivolumab. It is particularly devastating for cancer survivor patients to lose their eyesight after that. We thus recommend that clinicians be aware that ocular toxicities should be considered after the complaint of side effects from their patients. Thereby, referral for eye screening after any irAEs is crucial to ensure early detection and appropriate management of these complications.

## Conclusions

Like immunotherapy drugs, novel cancer therapy can cause irAEs. The onset of ocular side effects can start as early as a few weeks after the initiation of drugs. Patients should be counselled about the potential implication of their vision and offered a complete ocular examination in cases of eye-related complaints following the initiation of immunotherapies.
